# Supplementary material for: Ab initio predictions for 3D structure and stability of single- and double-stranded DNAs in ion solutions
Source: PLoS Comput Biol. 2022 Oct 19;18(10):e1010501. doi: 10.1371/journal.pcbi.1010501 (PMC9621594; doi:10.1371/journal.pcbi.1010501)
Supplement: S1 Text — The force field of the present model, the melting temperature calculation for dsDNAs at low strand concentrations, and the additional figures (Figs A-E) and tables (Tables A-D). (PDF) [file pcbi.1010501.s001.pdf]

Supporting information for

Ab initio predictions for 3D structure and stability of single- and double-stranded DNAs in ion solutions

Zi-Chun Mu[1,2,¶] , Ya-Lan Tan[1,¶], Ben-Gong Zhang[1], Jie Liu[1], and Ya-Zhou Shi[1,*]

[1] *Research Center of Nonlinear Science, School of Mathematical & Physical Sciences, Wuhan Textile University, Wuhan, 430200, China*

[2] *School of Computer Science and Artificial Intelligence, Wuhan Textile University, Wuhan, 430200, China*

[¶]The two authors contributed equally to the work.

[*] yzshi@wtu.edu.cn

1. The energy function of the present model

The total potential energy of a DNA conformation with CG representation in the present model is composed of eight components:

$$U = U_b + U_a + U_d + U_{exc} + U_{bp} + U_{bs} + U_{cs} + U_{el}. \tag{S1}$$

The first three terms are bonded potential for virtual bonds U_b, bond angles U_a and dihedrals U_d, which are used to mimic the connectivity and the local geometry of DNAs, and their expression forms are as follows.

$$U_b = \sum_{bonds} K_b(r - r_0)^2\,; \tag{S2}$$

$$U_a = \sum_{angles} K_\theta(\theta - \theta_0)^2\,; \tag{S3}$$

$$U_d = \sum_{dihedrals} \{K_\varphi[1 - cos(\varphi - \varphi_0)] + \frac{1}{2}K_\varphi[1 - cos3(\varphi - \varphi_0)]\}, \tag{S4}$$

where K_b, K_θ, and K_φ represent the energy strength, and r_0, θ_0, and φ_0 are the distances and angles for virtual bonds, bond angles and dihedrals at energy minimum, respectively. The initial parameters of these three potentials were derived from the Boltzmann inversion of the corresponding atomistic distribution functions obtained by the statistical analysis on the experimental structures in the PDB (http://www.rcsb.org/pdb/home/home.do); see Table A in S1 Text for the PDB code list and Fig A in S1 Text for the statistical distributions. It should be pointed out that two sets of parameters $\text{Para}_{\text{helical}}$ and $\text{Para}_{\text{nonhelical}}$ were used in the present model; see Table B in S1 Text. Since the known DNA structures are generally double helices, the parameters, $\text{Para}_{\text{helical}}$, directly obtained from these structures could not be reasonable to describe DNA chains during folding processes. Based on our previous model for RNAs [1-3], we employed another set of parameters (i.e., $\text{Para}_{\text{nonhelical}}$) for bonded potentials (Eqs. S2-S3), the strengths of which are half of $\text{Para}_{\text{helical}}$, to well simulate DNA folding approximated as a free chain. That is, only the $\text{Para}_{\text{nonhelical}}$ is used in the folding process, while in the final structure refinement, the $\text{Para}_{\text{helical}}$ and $\text{Para}_{\text{nonhelical}}$ are applied for nucleotides in base pairs and in loops/single-stranded regions, respectively, in order to accurately depict the more standard geometry

of helical parts. Based on the initial parameters, we did simulations for four DNAs including two dsDNAs (PDB codes: 1agh, 3bse) and two ssDNAs (PDB codes: 1ac7, 1jve), and further adjusted the parameters according to the comparisons between the simulated and experimental bond length/angle distributions [4,5]. The final parameters of bonded potentials are shown in Table B in S1 Text.

U_{exc} in Eq. S1 uses a pure Lennard-Jones potential to strictly limit excluded volume interactions between two nonbonded beads:

$$U_{exc} = \sum_{i<j}^{N} \begin{cases} 4\varepsilon \left[\left(\frac{\sigma_0}{r_{ij}}\right)^{12} - \left(\frac{\sigma_0}{r_{ij}}\right)^{6} \right] & if\ r_{ij} \le \sigma_0 \\ 0 & if\ r_{ij} > \sigma_0 \end{cases}, \quad \text{(S5)}$$

where ε = 0.26 kcal/mol is the interaction strength [1], σ_0 is the sum of the radii of bead i and j, and r_{ij} is the distance between bead i and j.

U_{bp} in Eq. S1 is the base-pairing interaction between bases in the canonical Watson-Crick base pairs (G-C and A-T). The potential is given by [1,6]

$$U_{bp} = \sum_{i<j-3}^{N_{bp}} \frac{\varepsilon_{bp}}{1 + k_{NN}\left(r_{N_iN_j} - r_{NN}\right)^2 + k_{CN}\sum_{i(j)}\left(r_{C_iN_j} - r_{CN}\right)^2 + k_{PN}\sum_{i(j)}\left(r_{P_iN_j} - r_{PN}\right)^2}, \quad \text{(S6)}$$

where ε_{bp} is the interaction strength $\varepsilon_{AT} = 2\varepsilon_{GC}/3$. r_{NN}, r_{CN}, and r_{PN} are three distances between the corresponding atoms of P, C and N in two paired nucleotides to describe the orientation of hydrogen-bonding interactions, and the values of them were obtained from the pairing bases in the PDB structures; see Fig C in S1 Text. k_{NN}, k_{CN} and k_{PN} in Eq. S6 are the corresponding energy strength.

U_{bs} in Eq. S1 is the base-stacking interaction between two nearest neighbour base pairs, and the energy is given by

$$U_{bs} = \frac{1}{2}\sum_{i,j}^{N_{st}} |G_{i,i+1,j-1,j}| \left\{ \left[5\left(\frac{\sigma_{st}}{r_{i,i+1}}\right)^{12} - 6\left(\frac{\sigma_{st}}{r_{i,i+1}}\right)^{10} \right] + \left[5\left(\frac{\sigma_{st}}{r_{j,j-1}}\right)^{12} - 6\left(\frac{\sigma_{st}}{r_{j,j-1}}\right)^{10} \right] \right\}, \quad \text{(S7)}$$

where σ_{st} is the optimum distance of two neighbour bases in the helix parts in PDB structures; see Fig C in S1 Text. $G_{i,i+1,j-1,j}$ in Eq. S7 is the strength of base-stacking energy and can be estimated by:

$$G_{i,i+1,j-1,j} = \Delta H - T(\Delta S - \Delta S_c), \quad \text{(S8)}$$

where ΔH and ΔS are the DNA thermodynamic parameters derived from experiments [7,8]. T is the

absolute temperature in Kelvin, and ΔS_c is the conformational entropy change which is naturally included in the Monte Carlo (MC) algorithm used in the present model due to the formation of one base pair stacking. The ΔS_c was calculated from MC simulations for an A-form double-stranded DNA, as shown in Fig D in S1 Text. In the simulations, we fixed the entire molecule except for nucleotides $\leqslant i$ or $\geqslant j$ and counted the number Ω of conformations which satisfy the condition of the stacking between base pairs (i, j) and (i+1, j-1) in the absence of the base-pairing and base-stacking constraints. Based on these, the conformational entropy changes for the formation of base stacking between base pairs (i, j) and (i+1, j-1) is calculated by

$$\Delta S_c = k_B \ln(\Omega/\Omega_0), \tag{S9}$$

where k_B is the Boltzmann constant, and Ω_0 is the total number of conformations searched in this simulation. As shown in Fig D in S1 Text, ΔS_c changes very slightly at different base pair location i. Consequently, for simplicity, the average value of -11.5 eu was used in the present model.

U_{cs} in Eq. S1 is the coaxial-stacking between the interfaced base-pairs of two discontinuous neighbour stems, and the energy given by:

$$U_{cs} = \frac{1}{2}\sum_{i-j,k-l}^{N_{cst}} \left|G_{i,k,l,j}\right| \{\left[1 - e^{-a(r_{ik}-r_{cs})}\right]^2 + \left[1 - e^{-a(r_{jl}-r_{cs})}\right]^2 - 2\}\,, \tag{S10}$$

where $G_{i,k,l,j}$ is the sequence-dependent base-stacking strength, approximate as the stacking strength between the corresponding nearest-neighbour base-pairs in an uninterrupted helix [7-9]. r_{ik} (or r_{jl}) is the distance between two interfaced bases i(j) and k(l) of two stems and a represents the extent of distance constraint. r_{cs} is the optimum distance between two coaxially stacked stems, and since the known structures with bulge/internal loops are very limited, here we referred to the statistical results of RNAs; see refs. 2 and 3.

The last term U_{el} in Eq. S1 is the electrostatic interaction between phosphates with reduced charges given by the Debye-Hückel approximation [1,6,10]:

$$U_{el} = \sum_{i<j}^{N_P} \frac{(Qe)^2}{4\pi\varepsilon_0\varepsilon(T)r_{ij}} \mathrm{e}^{-\frac{r_{ij}}{l_D}}. \tag{S11}$$

where e is the elementary charge and N_P is the number of phosphate beads in a DNA chain. l_D is Debye length to define the ionic screening and can be calculated through $l_D = (\frac{\varepsilon_0\varepsilon(T)k_BT}{2N_Ae^2I})^{1/2}$, where I is the ionic strength which depends on the concentration and charge number of ions, and $\varepsilon(T)$ is an

effective dielectric constant of water [1-3,10]. Based on the counterion condensation theory [10] and the tightly bound ion model [11,12], the reduced charge fraction Q could be written as $\mathrm{Q} = \frac{b}{v l_B}$ for pure ion monovalent (v=1) or divalent (v=2) ion solutions and $Q = f_{\mathrm{Na}^+}\left(\frac{b}{l_B}\right) + (1 - f_{\mathrm{Na}^+})\left(\frac{b}{2l_B}\right)$ for mixed Na^+/Mg^{2+} solutions, where b is the charge spacing on DNA backbone, l_B is the Bjerrum length, f_{Na^+} and 1-f_{Na^+} are the contribution fraction from Na^+ and Mg^{2+}, respectively. The empirical formula

$$f_{\mathrm{Na}^+} = \frac{[\mathrm{Na}^+]}{[\mathrm{Na}^+] + x[\mathrm{Mg}^{2+}]} \qquad (S12)$$

derived by the tightly bound ion model is used for mixed divalent/monovalent ion solutions [11,12], and $x = (8.1 - 32.4/N)(5.2 - \ln[\mathrm{Na}^+])$. $[Na^+]$ and $[Mg^{2+}]$ are the corresponding concentrations in molar (M) and N is the chain length in *bp*.

The parameters of the above described potentials are listed in Table B in S1 Text, which were derived through the statistical analysis on the known structures and the comparisons between the predictions by the model and the experimental data/structures.

2. Melting temperature calculations for dsDNAs with low strand concentrations

To improve the simulation efficiency for dsDNA with low strand concentrations c_s (e.g., <0.1mM), we performed the MC simulations at a relatively high strand concentration $c_s{}^h$ (e.g., 1mM) [3]. Since simple dsDNAs used in this work generally transform between two states (folded and unfolded) as temperature increases, we employed traditional two-state model to fit the fractions of unfolded state (i.e., $1 - f_F(\mathrm{T})$); see Eqs. 4-6 in the main text. That is, the fraction of the number of denatured base pairs at $c_s{}^h$ can be given by

$$f(\mathrm{T}; c_s{}^h) = 1 - \frac{1}{1 + e^{\left(\mathrm{T} - \mathrm{T_m}(c_s{}^h)\right)/\mathrm{dT}}}, \qquad (S13)$$

where $\mathrm{T_m}(c_s{}^h)$ is the corresponding melting temperature. Based on the refs. 13 and 14, the fraction ($f(\mathrm{T}; c_s)$) of denatured base pairs at any other strand concentration c_s, especially a lower strand concentration, can be calculated by

$$f(\mathrm{T}; c_s) = \frac{\lambda f(\mathrm{T}; c_s{}^h)}{1 + (\lambda - 1) f(\mathrm{T}; c_s{}^h)}, \qquad (S14)$$

where $\lambda = {c_s}^h/c_s$. By substituting Eq. S13 into Eq. S14, $f(\mathrm{T}; c_s)$ can be rewritten as

$$f(\mathrm{T}; c_s) = \frac{\lambda}{\lambda + e^{-\left(\mathrm{T}-\mathrm{T_m}\left({c_s}^h\right)\right)/\mathrm{dT}}}. \qquad (S15)$$

Therefore, if we set $f(\mathrm{T}; c_s)$ equals to 0.5, the temperature T in Eq. S15 will be the melting temperature $T_m(c_s)$ of the dsDNA at the strand concentration of c_s:

$$T_m(c_s) = T = T_m(c_s) - dTln\lambda. \qquad (S16)$$

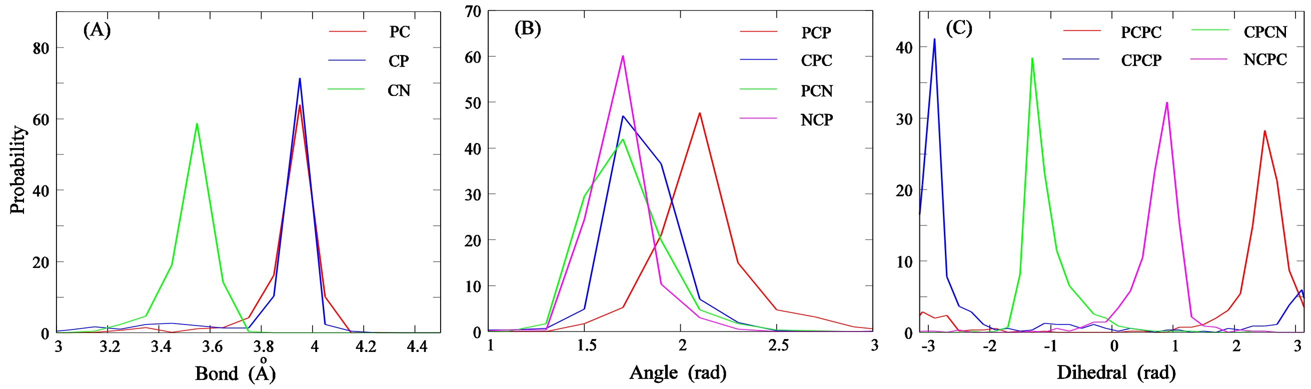

Fig A. The normalized probability distributions of (A) the virtual bond lengths (PC, CP and CN), (B) bond angles (PCP, CPC, PCN and NCP), and (C) dihedrals (PCPC, CPCP, CPCN and NCPC) in known structures listed in Table A in S1 Text.

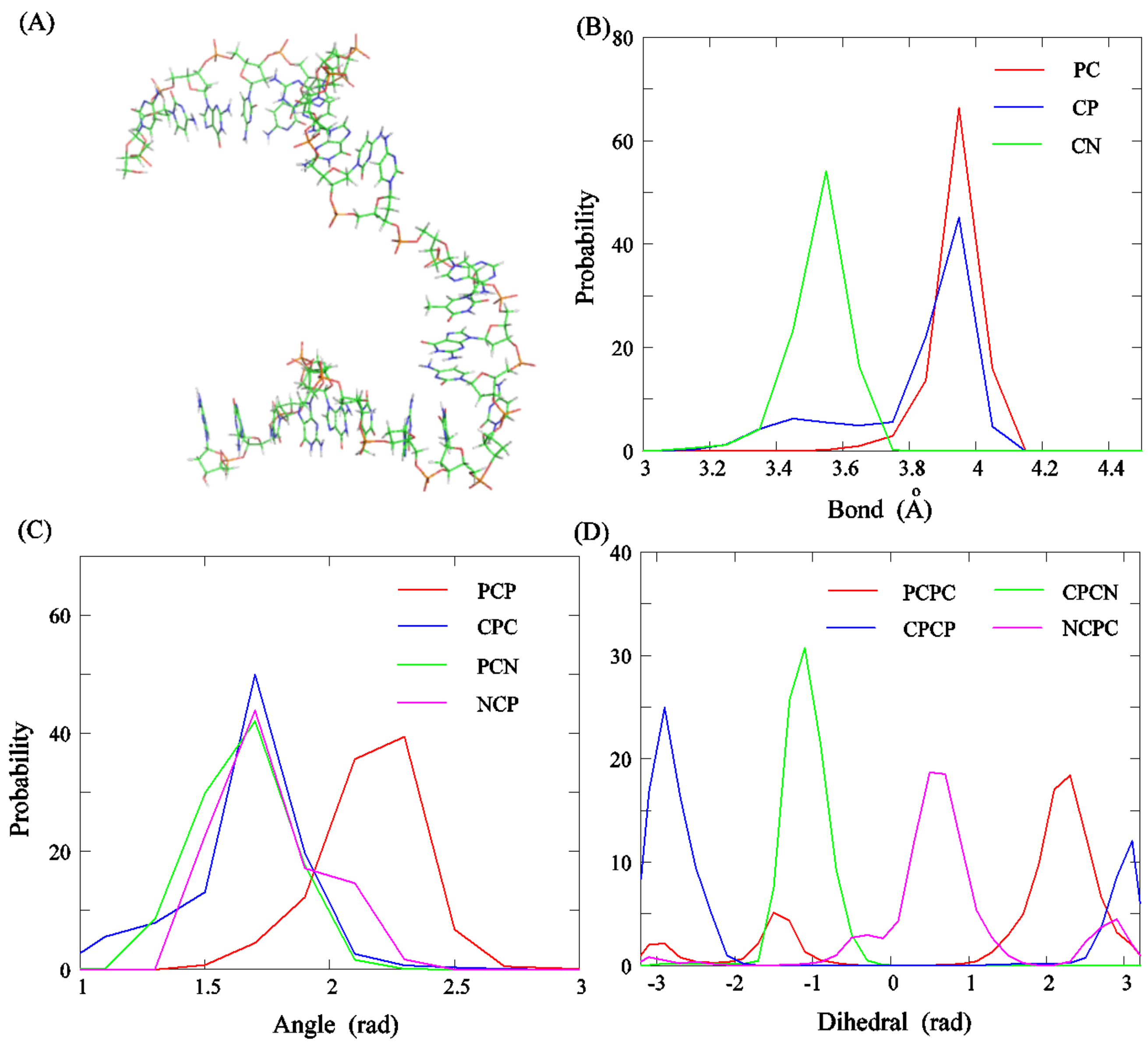

Fig B. (A) A snapshot for a DNA conformation from a short-time (10ns) MD simulation of a random sequence (5'-CTGCCACGCCATGCCTGTGACGA-3'). (B-D) The normalized probability distributions of (B) the virtual bond lengths (PC, CP and CN), (C) bond angles (PCP, CPC, PCN and NCP), and (D) dihedrals (PCPC, CPCP, CPCN and NCPC) in conformations from the MD simulation. The MD simulation was performed in the isothermic-isobaric ensemble (P =1 atm, T=298 K) using the Gromacs 4.6 software package with AMBER ff99bsc0 force fields and TIP3P water model [15,16]. The counterions of Na^+ and the salt of 1M NaCl were added to ensure that the simulated systems are fully neutralized. Although we tried to extract the bonded parameters based on (B-C), we gave them up due to the differences in optimum values of several angles (e.g., PCP and PCPC) between experimental and MD simulated structures; see Fig A in S1 Text.

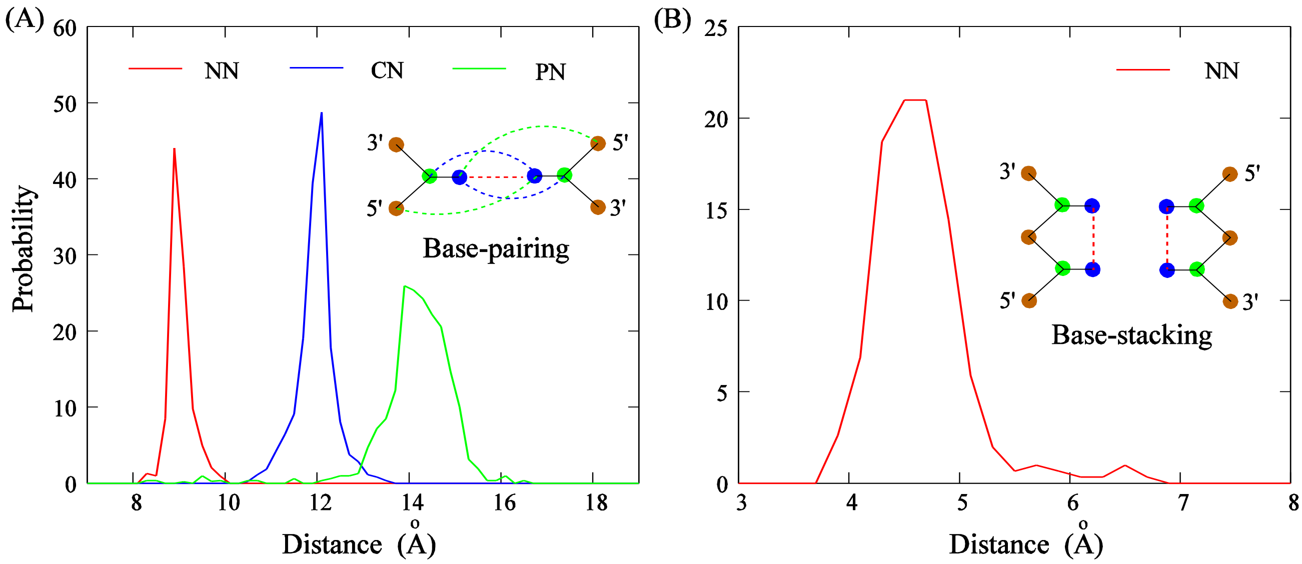

Fig C. The normalized probability distributions of distances for (A) base-pairing and (B) base-stacking shown in Fig 1B in main text, which are obtained by the statistical analysis of the stems over the known structures listed in Table A in S1 Text.

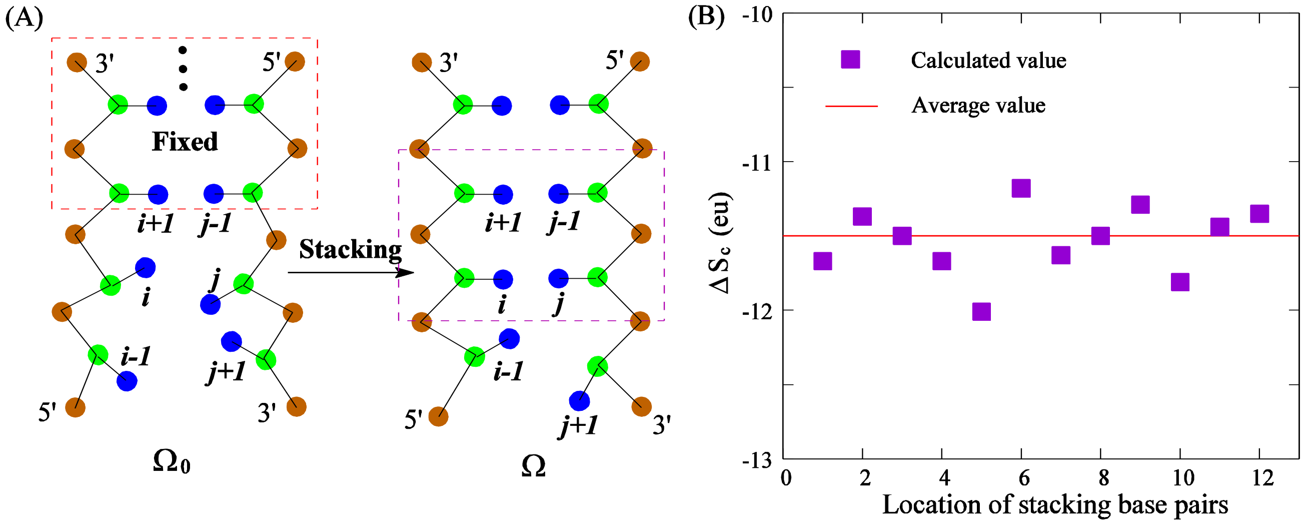

Fig D. The illustration for the calculation of the conformational entropy changes ΔS_c (in Eq. S8) of base-stacking formation naturally included in our MC simulations. (A) The schematic diagram for the formation of one base-stacking between base pairs (*i*, *j*) and (*i*+1, *j*-1). The dashed box in left structure schematic represents the part fixed in the simulations, and that in right schematic shows the formation of the stacking between base pairs (*i*, *j*) and (*i*+1, *j*-1). Ω_0 means the number of all possible conformations searched in the simulation, and Ω is the number of conformations with the stacking between base pairs (*i*, *j*) and (*i*+1, *j*-1). (B) The conformational entropy changes ΔS_c for the formation of base-pairs stacking at different location *i* (symbols), and the average value (line).

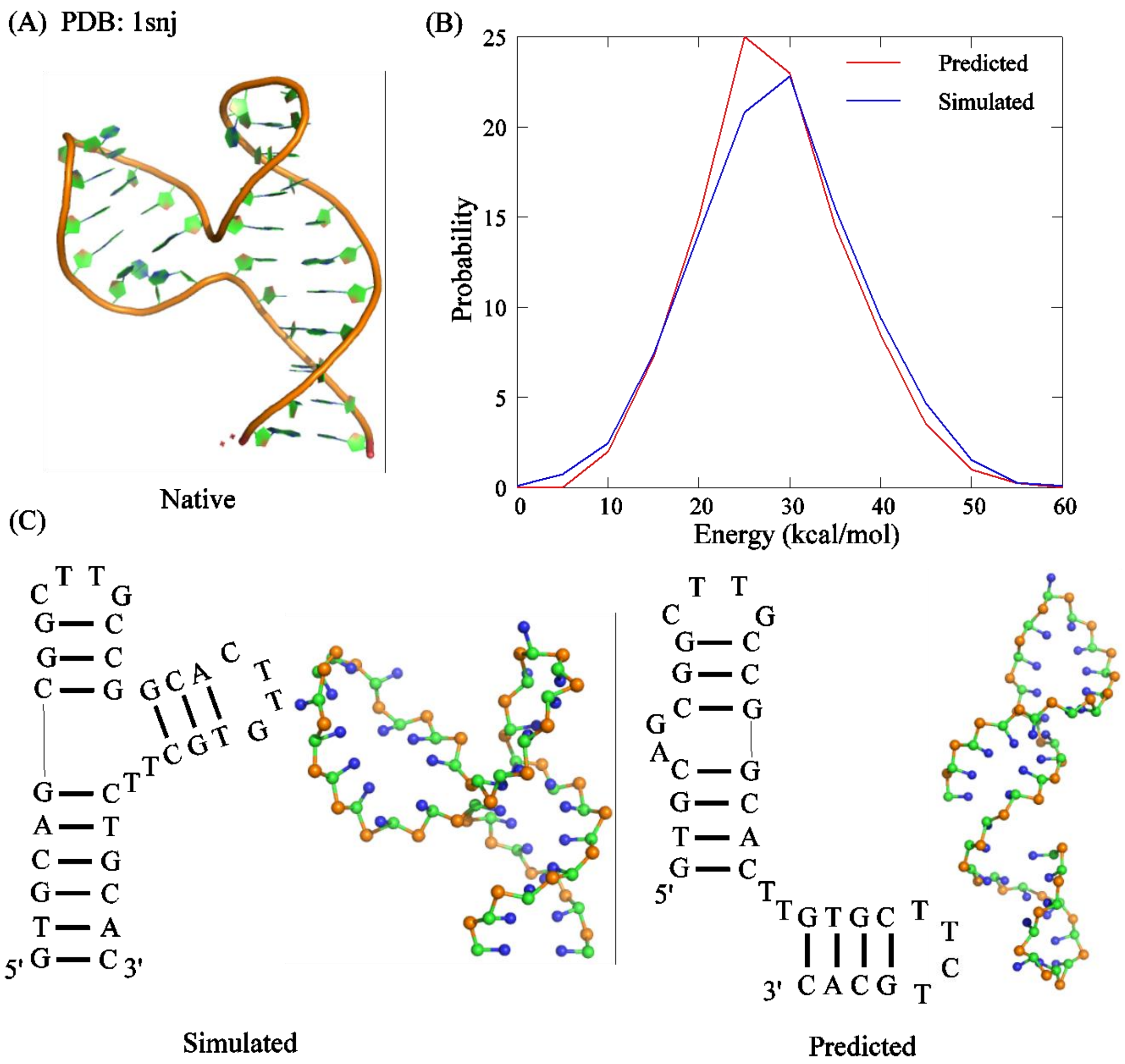

Fig E. In this work, we have tried unsuccessfully to predict 3D structure for one DNA three-way junction using the present model. To figure out the possible reason of the failure, we further employed the present model to perform another simulation for the junction at room temperature taking the native structure as input (i.e., the initial conformation), and made comparison between energies of simulated conformations and predicted ones at the same temperature. (A) The native tertiary structure of the DNA junction (PDB: 1snj) using in the work [17]. (B) The normalized probability distributions of energies from (C) predicted (right) and simulated (left) conformations, respectively, and we found that there is no significant difference of energy between two different ensembles of conformation.

Table A. The PDB codes of 138 DNAs[a] used in our statistical analysis for CG force field.

1ag5	1agk	1aul	1aw4	1bdz	1bdn	1b6x	1bub	1cvx	1cs7
1dxa	1dnm	1dcr	1d13	1d16	1d49	1d63	1d89	1db6	1eek
1ezn	1en1	1en3	1fyk	1fv8	1g6d	1i0f	1juu	1lai	1lp7
1l0r	1la8	1noq	1puy	1qdk	1qph	1qe7	1snj	1sk5	1wqy
1zfb	1zfe	1zfg	1zfh	1zyf	1zyg	1zew	1zf9	107d	116d
119d	126d	158d	183d	195d	196d	2arg	2b1b	2b1d	2d47
2f1q	2gyx	2kuz	2k0v	2k67	2k68	2k69	2lbi	2lgm	2lzv
2lzw	2lsc	2ll9	2l13	2mav	2mci	2miv	2mjj	2mnf	2m2c
2npw	2neo	2org	2pik	2rrr	2rvp	2rt8	238d	240d	260d
272d	285d	287d	3co3	3gsj	3l1q	3omj	3qsc	3qk4	3r86
3v06	307d	339d	348d	363d	4e7y	4f8g	4j2i	4kbd	414d
424d	440d	5ewb	5gun	5ip8	5ju4	5j3g	5ki4	5mvp	5mvq
5m68	5uzf	5xuv	6asf	6ast	6dm7	6dy5	6g8s	6iyq	6ror
6rou	6s7d	7b4z	7edw	7kcl	7vck	7ril	7sb8		

[a] Note that the DNAs used for parameter determination are with no overlap with ssDNAs/dsDNAs used for model validation on 3D structure prediction.

Table B. The parameters of potentials in Eqs. S2-S10

Bond length U_b			
	K_b (kcal/mol/Å^2)		r_0 (Å)
	Para$_{helical}$ [a]	Para$_{nonhelical}$ [b]	Para$_{helical}$ [a]/Para$_{nonhelical}$ [b]
P_iC_i	196.4	98.2	3.95
C_iP_{i+1}	141.0	70.5	3.95
C_iN_i	91.6	45.8	3.55
Bond angle U_a			
	K_θ (kcal/mol/rad^2)		θ_0 (rad)
	Para$_{helical}$ [a]	Para$_{nonhelical}$ [b]	Para$_{helical}$ [a]/Para$_{nonhelical}$ [b]
$P_iC_iP_{i+1}$	19.6	9.8	2.1
$C_{i-1}P_iC_i$	17.2	8.6	1.8
$P_iC_iN_i$	13.0	6.5	1.7
$N_iC_iP_{i+1}$	28.6	14.3	1.7
Bond dihedral U_d			
	K_φ (kcal/mol/rad^2)		φ_0 (rad)
	Para$_{helical}$ [a]	Para$_{nonhelical}$ [b]	Para$_{helical}$ [a]/Para$_{nonhelical}$ [b]
$P_iC_iP_{i+1}C_{i+1}$	2.6	1.3	2.5
$C_{i-1}P_iC_iP_{i+1}$	8.0	4.0	-2.9
$C_{i-1}P_iC_iN_i$	6.4	3.2	-1.3
$N_{i-1}C_{i-1}P_iC_i$	3.2	1.6	0.9
Nonbonded			
r_{NN} (Å)	8.9	k_{NN} (kcal/mol/Å^2)	2.66
r_{CN} (Å)	12.1	k_{CN} (kcal/mol/Å^2)	1.37
r_{PN} (Å)	14.1	k_{PN} (kcal/mol/Å^2)	0.46
σ_{st} (Å)	4.5	ε_{bp} (kcal/mol) [c]	-2.2/-3.5
r_{cs} (Å)	4.9	a (kcal/mol//Å)	0.4

[a] The Para$_{helical}$ are only used in the processes of folded structure refinement for the base-pairing regions (stems) in the initially folded structure.

[b] The Para$_{nonhelical}$ are used in DNA folding processes to possibly describe DNAs as free chains.

[c] For dsDNAs, ε_{bp} is equal to -2.2kcal/mol, while for ssDNAs, ε_{bp} takes -3.5kcal/mol.

Table C. Double-stranded DNAs structure prediction at 1 M $[Na^+]$

dsDNAs	PDB	Sequence [a]	Length (nt)	Type of structure[b]	$RMSD_{mean}$ (Å)[c]	$RMSD_{min}$ (Å)[d]	$RMSD_{3dRNA/DNA}$(Å)[e]
1	2n5p	ATGGAGCTC/GAGCTCCAT	18	D	2.2	1.1	2.3
2	1kvh	CCCGATGC/GCAATTCGGG	18	DB	3.5	2.3	5.4
3	158d	CCAAGCTTGG/CCAAGCTTGG	20	D	2.4	1.3	2.1
4	1agh	CGGACAAGAAG/CTTCTTGTCCG	22	D	2.0	0.8	2.6
5	109d	CGCGAATTCGCG/ CGCGAATTCGCG	24	D	2.5	1.6	3.2
6	424d	ACCGACGTCGGT/ ACCGACGTCGGT	24	D	2.5	1.5	2.5
7	1bna	CGCGAATTCGCG/ CGCGAATTCGCG	24	D	2.2	1.2	2.3
8	141d	AGCTTGCCTTGAG/ CTCAAGGCAAGCT	26	D	3.2	1.6	5.2
9	1p96	GTCCGATGCGTG/ CACGCAGTTCGGAC	26	DB	3.1	1.9	8.3
10	1mnn	TGCGACACAAAAAC/ AGTTTTTGTGTCGC	28	DD	3.1	1.7	1.8
11	1qsk	CGTAGCCGATGC/ GCATCGAAAAAGCTACG	29	DB	3.2	1.8	3.5
12	3kbd	CTGCTCACTTTCCAGG/ CCTGGAAAGTGAGCAG	32	D	2.9	1.9	3.2
13	1saa	CATGTGACGTCACATG/ CATGTGACGTCACATG	32	D	3.6	2.0	3.7
14	1ir5	CACTACTCTTTGTAGTG/ CACTACAAAGAGTAGTG	34	D	2.9	1.8	2.2
15	1tqr	GGAAAATCTCTAGCAGT/ ACTGCTAGAGATTTTCC	34	D	3.5	2.2	3.1
16	3bse	ACACTACAATGTTGCAAT/ GTATTGCAACATTGTAGT	36	DD	2.6	1.6	1.9
17	1lmb	AATACCACTGGCGGTGATAT/ TATATCACCGCCAGTGGTAT	40	DD	3.3	2.0	2.8
18	2jyk	ACAGCTTATCATCGATCACGT/ ACGTGATCGATGATAAGCTGT	42	D	4.8	3.3	4.9
19	5t1j	AATTTCACACCTAGGTGTGAAATT/ AATTTCACACCTAGGTGTGAAATT	48	D	5.0	3.4	3.9
20	1mnm	GATTACCTAATAGGGAAATTTACA CG/CCGTGTAAATTTCCCTATTAGG TAAT	52	DD	5.3	2.8	2.0

[a] The sequences of two strands are separated by '/', both start from 5', and the unpaired nucleotides are underlined. [b] D: double-helix, DB: double-helix with bulge loop, DD: double-helix with dangling ends. [c,d] The mean/minimum RMSD calculated over CG beads of structures predicted by the present model from the corresponding atoms of the native structure. [e] The mean RMSDs of top 5 structures predicted by the 3dRNA/DNA [18,19] from the native structure.

Table D. Single-stranded DNAs structure prediction at 1 M [Na^+]

ssDNAs	PDB ID	Sequence[a]	Length (nt)	Type of structure[b]	$RMSD_{mean}$ (Å)[c]	$RMSD_{min}$ (Å)[d]	$RMSD_{3dRNA/DNA}$ (Å)[e]
1	1kr8	GCGAAGC	7	H	1.9	1.0	2.5
2	2k71	GCGAAAGC	8	H	2.2	1.1	3.3
3	2lo8	GCCGCAGTGC	10	HB	2.4	1.1	3.4
4	1bjh	GTACAAAGTAC	11	H	2.5	1.3	3.7
5	2lo5	GGCCGCAGTGCC	12	HB	3.3	2.2	4.1
6	1p0u	GCATCGACGATGC	13	H	2.1	1.1	3.1
7	2m8y	CGCGAAGCATTCGCG	15	H	2.2	1.2	2.8
8	1ac7	ATCCTAGTTATAGGAT	16	H	2.1	1.0	3.2
9	1xue	GTGGAATGCAATGGAAC	17	HI	4.3	2.2	3.0
10	1ii1	AGGATCCUTTTGGATCCT	18	H	3.3	1.8	3.5
11	1dgo	AGGATCCTUTTGGATCCT	18	H	3.3	2.0	3.4
12	4kb1	GGCCCTCTTTAGGGCCTC	18	HB	3.9	2.7	3.9
13	1ecu	GCGCGAAACTGTTTCGCGC	19	H	3.5	1.6	2.5
14	2l5k	CAGTTGATCCTTTGGATACCCTG	23	HI	4.8	2.8	3.5
15	2oey	CCATCGTCTACCTTTGGTAGGATGG	25	HB	5.0	2.9	4.8
16	1jve	CCTAATTATAACGAAGTTATAATTAGG	27	H	3.1	1.6	2.6
17	1ngo	CTCTTTTGTAAGAAATACAAGGAGAG	27	HI	5.3	3.3	4.0
18	6u82	GCTAATCTAATCAACCGCAGGTTGATTAGCCCATTAGC	38	HB	4.8	2.5	1.9
19	6x68_1	TTACGCAGACTATCTTTCTAGGGTTAACCCTAGAAAGATAGTCTGCGTAA	50	H	4.1	2.8	9.2
20	6x68_2	CATGCGTCAATTTTACGCAGACTATCTTTCTAGGGTTAACCCTAGAAAGATAGTCTGCGTAAAATTGACGCATG	74	H	5.6	3.2	11.6

[a] The sequences start from 5’, and the loop nucleotides are underlined. [b] H: hairpin, HB: hairpin with bulge loops, HI: hairpin with internal loops. [c,d] The mean/minimum RMSD calculated over all CG beads of structures predicted by the present model from the corresponding atoms of the native structures. [e] The mean RMSDs of top 5 structures predicted by the 3dRNA/DNA [18,19] from the native structure.

Supporting References

1. Shi YZ, Wang FH, Wu YY, Tan ZJ. A coarse-grained model with implicit salt for RNAs: predicting 3D structure, stability and salt effect. J Chem Phys. 2014;141: 105102.
2. Shi YZ, Jin L, Feng CJ, Tan YL, Tan ZJ. Predicting 3D structure and stability of RNA pseudoknots in monovalent and divalent ion solutions. PLoS Comput Biol. 2018;14: e1006222.
3. Jin L, Shi YZ, Feng CJ, Tan YL, Tan ZJ. Modeling structure, stability, and flexibility of double-stranded RNAs in salt solutions. Biophys J. 2018;115: 1403-1416.
4. Sun T, Minhas V, Korolev N, Mirzoev A, Lyubartsev AP, Nordenskiold L. Bottom-up coarse-grained modeling of DNA. Front Mol Biosci. 2021;8: 645527.
5. Leonarski F, Trovato F, Tozzini V, Les A, Trylska J. Evolutionary algorithm in the optimization of a coarse-grained force field. J Chem Theory Comput. 2013;9: 4874-4889.
6. Chakraborty D, Hori N, Thirumalai D. Sequence-dependent three interaction site model for single- and double-stranded DNA. J Chem Theory Comput. 2018;14: 3763-3779.
7. SantaLucia J, Jr., Allawi HT, Seneviratne PA. Improved nearest-neighbor parameters for predicting DNA duplex stability. Biochemistry. 1996;35: 3555-3562.
8. SantaLucia J, Jr., Hicks D. The thermodynamics of DNA structural motifs. Annu Rev Biophys Biomol Struct. 2004;33: 415-440.
9. Peyret N, Seneviratne PA, Allawi HT, SantaLucia J, Jr. Nearest-neighbor thermodynamics and NMR of DNA sequences with internal A.A, C.C, G.G, and T.T mismatches. Biochemistry. 1999;38: 3468-3477.
10. Manning GS. The molecular theory of polyelectrolyte solutions with applications to the electrostatic properties of polynucleotides. Q Rev Biophys. 1978;11: 179-246.
11. Tan Z, Zhang W, Shi Y, Wang F. RNA folding: structure prediction, folding kinetics and ion electrostatics. Adv Exp Med Biol. 2015;827: 143-183.
12. Tan ZJ, Chen SJ. Electrostatic correlations and fluctuations for ion binding to a finite length polyelectrolyte. J Chem Phys. 2005;122: 44903.
13. Ouldridge TE, Louis AA, Doye JP. Extracting bulk properties of self-assembling systems from small simulations. J Phys Condens Matter. 2010;22: 104102.
14. Privalov PL, Crane-Robinson C. Translational entropy and DNA duplex stability. Biophys J. 2018;114: 15-20.
15. Bao L, Zhang X, Shi YZ, Wu YY, Tan ZJ. Understanding the relative flexibility of RNA and DNA duplexes: stretching and twist-stretch coupling. Biophys J. 2017;112: 1094-1104.
16. Zhang C, Tian F, Lu Y, Yuan B, Tan ZJ, Zhang XH, et al. Twist-diameter coupling drives DNA twist changes with salt and temperature. Sci Adv. 2022;8: eabn1384.

17. Wu B, Girard F, van Buuren B, Schleucher J, Tessari M, Wijmenga S. Global structure of a DNA three-way junction by solution NMR: towards prediction of 3H fold. Nucleic Acids Res. 2004;32: 3228-3239.
18. Zhang Y, Xiong Y, Xiao Y. 3dDNA: A computational method of building DNA 3D structures. Molecules. 2022;27: 5936.
19. Zhang Y, Wang J, Xiao Y. 3dRNA: 3D structure prediction from linear to circular RNAs. J Mol Biol. 2022;434: 167452.
